# Sustainable potential of shallow geothermal heat recycling in Dresden, Germany

**DOI:** 10.1098/rsta.2025.0013

**Published:** 2025-11-06

**Authors:** Verena Dohmwirth, Kathrin Menberg, Peter Bayer, Matthias Mauder, Philipp Blum, Susanne Benz

**Affiliations:** ^1^ Institute of Photogrammetry and Remote Sensing, Karlsruhe Institute of Technology, Karlsruhe, Baden-Württemberg, Germany; ^2^ Applied Geosciences, Karlsruhe Institute of Technology, Karlsruhe, Baden-Württemberg, Germany; ^3^ Applied Geology, Martin-Luther-Universität Halle-Wittenberg, Halle (Saale), Thüringen, Germany; ^4^ Intitute of Hydrology and Meteorology, Technische Universität Dresden, Dresden, Saxony, Germany

**Keywords:** technical geothermal potential, sustainable supply rate, fair carbon price

## Abstract

In this study, we investigate the geothermal potential of the shallow subsurface in Dresden, Germany. The analysis considers the *status quo* scenario in which accumulated heat can be recycled. Installing all possible geothermal systems based on the available space, this heat could supply Dresden’s residents for 3 years with energy for space heating. However, a fair CO_2_ price would have to be implemented to improve economic value. Next, a near-future scenario is studied, in which accumulated heat has been *recycled* and, considering all technical constraints, the annual heat input provides a sustainable potential that can provide up to 4.5% of annual heating demands (HDs). However, there is a very high spatial variability that is studied in regard to its socio-economic implications. Finally, two far-future scenarios (*SSP245* and *SSP585*) are studied to understand the effect of climate change on the suitability of geothermal systems. Depending on the scenario and circumstances, up to 82% of the city’s climate neutrality targets might be reached.

This article is part of the theme issue ‘Urban heat spreading above and below ground’.

## 1. Introduction

In 2023, 67% of the residential energy consumption in Germany was used for space heating, while approximately 15% of this energy was supplied by renewable sources [1]. Thus, residential heating demand (HD) contributes significantly to ${\text{CO}}_{2}$ emissions, which exacerbate climate change and lead to higher temperatures in air, water and soil. Consequently, one of the most crucial climate protection measures is the reduction of ${\text{CO}}_{2}$ emissions for residential heating [2]. At the same time, the urban environment is severely overheating and urban heat islands are formed, particularly in densely built areas [3]. This effect is also observed underground (e.g. [4]), where the anthropogenic heat losses such as from underground infrastructure [5,6] and elevated natural heat fluxes from sealed surfaces or climate change [7–9] contribute to warming of shallow aquifers. Changing temperatures are shown to affect groundwater quality [10], ecology [11] and river habitats [12]. In addition, heat transport to the surface from local groundwater temperature (GWT) extremes, particularly those shaped by underground infrastructures [13], has the potential to affect local heat fluxes in the atmosphere [14]. See table 1 for nomenclature.

**Table 1.** Nomenclature.

ΔT	temperature difference
λ	thermal conductivity
*A*	area
*a*	year
BHE	borehole heat exchanger
$c_{v}$	heat capacity
CMIP6	Coupled Model Intercomparison Project
COP	coefficient of performance
$D_{built}$	mean basement depth below ground
DH	district heating
$E_{CO_{2},elec}$	$CO_{2}$ emission per kWh of electricity
$E_{CO_{2},gas}$	$CO_{2}$ emission per kWh of gas
$f_{CO_{2},elec}$	reduce of $CO_{2}$ emission in power mix
$f_{elec}$	average electricity price development
$f_{gas}$	average gas price development
GH	gas heater
GSHP	ground source heat pump
GST	ground surface temperature
GW	groundwater
GWT	groundwater temperature
$I_{gas}$	invest for gas heater
$I_{geo}$	invest for heat pump
*l*	probe length
*n*	number of possible installation area
$P_{CO_{2}}$	$CO_{2}$ emission costs
$P_{DH}$	median net heat loss
$P_{elec}$	electricity price
$P_{gas}$	gas price
*Q*	heat flow
$Q_{accumulated}$	accumulated heat
$Q_{annual}$	annual heat flow
*q*	heat flux
$q_{BHE}$	heat extraction rate in 100 m depth
$q_{built}$	heat flux by built-up area
$q_{DH}$	heat flux by district heating network
$q_{dw}$	mode heat loss directed downwards
$q_{surf}$	heat flux by open surface area
$q_{tot}$	total heat flux directed
$q_{tunnel}$	heat flux by tunnels
$q_{uw}$	heat flux directed upwards
$r_{DH}$	net heat loss directed downwards
*S*	technical exploitation supply rate
SPF	seasonal performance factor
SSP	shared socio-economic pathway
SubSUHI	subsurface urban heat island
$SusS_{BHE}$	technical sustainable supply rate
*T*	temperature
$T_{air}$	air temperature
$T_{built}$	mean basement temperature
$T_{GW}$	groundwater temperature
$T_{surf}$	surface temperature
*t*	operating time

Harnessing the accumulated heat of subsurface urban heat islands (SubSUHIs) through shallow geothermal energy systems such as ground source heat pumps (GSHPs) presents an opportunity to lower ${\text{CO}}_{2}$ emissions in local heating markets while also mitigating subsurface thermal pollution [15]. In our analysis, we consider closed-loop GSHPs which harness geothermal energy via borehole heat exchangers (BHEs) for heating purposes. The efficiency of a heat pump is described by the coefficient of performance (COP), i.e. the ratio between the heat output and the electrical power consumed:


(1.1) $$COP=\frac{P_{heat}}{P_{elec}}.$$

The COP is measured under standardized and ideal conditions and does not take into account real operation. Therefore, the seasonal performance factor (SPF) is introduced to account for actual operation over the course of the year with fluctuating temperatures and operating conditions. The SPF is around 4 for currently operated GSHPs [16].

Within an urban environment, geothermal systems for energy extraction are advantageous. For one, the initial extraction temperature is higher, allowing the GSHP to run more efficiently and for a longer time [17,18]. This is often quantified in the form of technical potentials [19], which consider technical constraints such as borehole depth and available drilling space, with the idea of exploitation, i.e. BHES are designed to extract heat for e.g. 50 years, cooling the soil to 0°C within that time [20]. Hence, we name this *technical exploitation*, i.e. the geothermal heat that can be technically extracted by GSHP. For example, Tissen *et al*. [21] calculated the supply rate of the technical exploitation for Vienna, identifying key locations for shallow geothermal use taking available space into account. However, one barrier to *technical exploitation* on a city-wide scale is the high investment costs of GSHP [22]. Hence, the expansion of geothermal energy use is progressing slowly [23] despite the introduction of political measures in Germany [24,25] and the economic advantage of lower operating costs compared to fossil fuels. Yet, it is not clarified whether the politically prescribed ${\text{CO}}_{2}$ pricing [26] is sufficient to make shallow geothermal energy competitive with fossil fuel heating systems in the long term. This information is essential for encouraging a shift towards sustainable energy sources and reaching the goals of the *Paris Agreement* [27].

Furthermore, the urban ground either already stores extra waste heat that can be extracted [28], or experiences a greater heat flow replenishment compared to rural areas [29]. Several studies have quantified the anthropogenic heat fluxes that contribute to the so-called *theoretical, sustainable* geothermal potential (e.g. [18,30,31]). They compute heat fluxes that sustainably replenish the reservoir, e.g. from the ground surface, buildings and underground infrastructures. With climate change, these heat fluxes will increase, thereby raising the possibility for sustainable heat recycling [32]. These studies discuss the theoretical potential of recycling the heat influx in place. Extracting this energy leaves subsurface temperatures unchanged and at natural, rural levels in the long term. However, despite these advances, the gap between theoretical estimates of shallow geothermal heat recycling (i.e. extracting only what is replenished each year from the surface) and practical implementations at a city-wide scale has not been bridged.

Here we introduce the *technical*, *sustainable* potential that describes the part of the *theoretical*, *sustainable* potential that can be extracted in a sustainable manner by GSHPs while considering technical and spatial limitations. A sustainable manner is here defined as (a) GSHPs first extract the accumulated heat and then (b) once the accumulated heat has been recycled, extract only an amount of heat equal to the heat influx in place that sustainably replenishes, i.e. often not running at full technical exploitation. This definition combines the concept of *technical potential* [19] and the idea of sustainability, meaning to only extract the sustainable extraction rate while technical and spatial limitations are considered.

Also, the societal aspects of shallow geothermal heat recycling have not yet been discussed in the context of distributional energy justice. That is the question of whether the benefits (such as access to affordable and/or green heat) of an energy source are distributed equally among the different socio-economic groups in our society, which is often not the case [33]. We analyse it here for the *technical, sustainable* potential, which promises long-lasting benefits.

In summary, in this study, we describe the potential for the implementation of GSHPs as the city-wide source for space heating in Dresden, Germany, focusing not only on its technical feasibility and sustainability in the form of waste heat recycling (i.e. extracting only the heat influx in place), but also on economic and social aspects.

## 2. Material and methods

### (a) Overview

**Figure 1.**
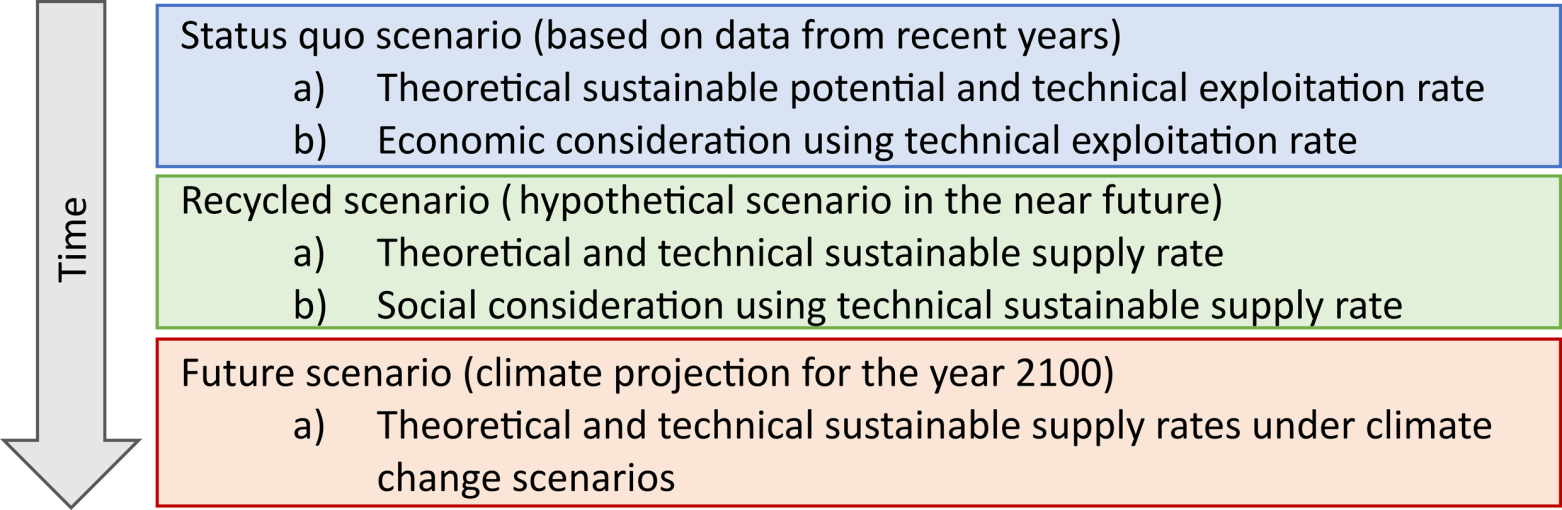
Systematic presentation of our workflow. Right now, the *status quo* is that waste heat has accumulated and can be recycled. Once it is *recycled,* the annual heat input may be used for sustainable shallow geothermal heat recycling. Under *climate change,* the annual heat input and hence the sustainable heat supply increases.

We consider the temporal change in shallow geothermal heat recovery based on three scenarios, as was done by Benz *et al*. [32] shown in figure 1. The scenarios are structured from present to near-future to far-future, moving downward along the time axis. First, we describe a *status quo* scenario: GWTs under the cities are elevated, and the accumulated heat can be harvested with shallow geothermal energy systems. We quantify the *theoretical sustainable potential* of the accumulated heat and the *technical exploitation* as heat supply rates, but we also study the economic (dis)advantages of space heating with geothermal energy in comparison to conventional space heating. Once the accumulated heat has been recycled and GWTs are reduced to their natural levels, we move into the near-future, *recycled* scenario. Here we can quantify the *technical sustainable* supply rate. That is, extracting only the heat that replenishes each year from heat transport into the shallow underground. We also study the societal effect of the long-term technical sustainable potential focusing on energy justice. Finally, two *far-future* scenarios are developed. Following the methodology in [32], we study the annual heat transport and *technical sustainable potential* for the year 2100 assuming GWT is kept at the current, natural levels as used in the *recycled* scenario. Through this analysis, we are able to describe a world where city-wide shallow geothermal heat recycling is implemented and where anthropogenic waste heat (as well as the heat transported into the underground from climate change) is reused instead of accumulating and contributing to potential thermal aquifer pollution.

**Figure 2.**
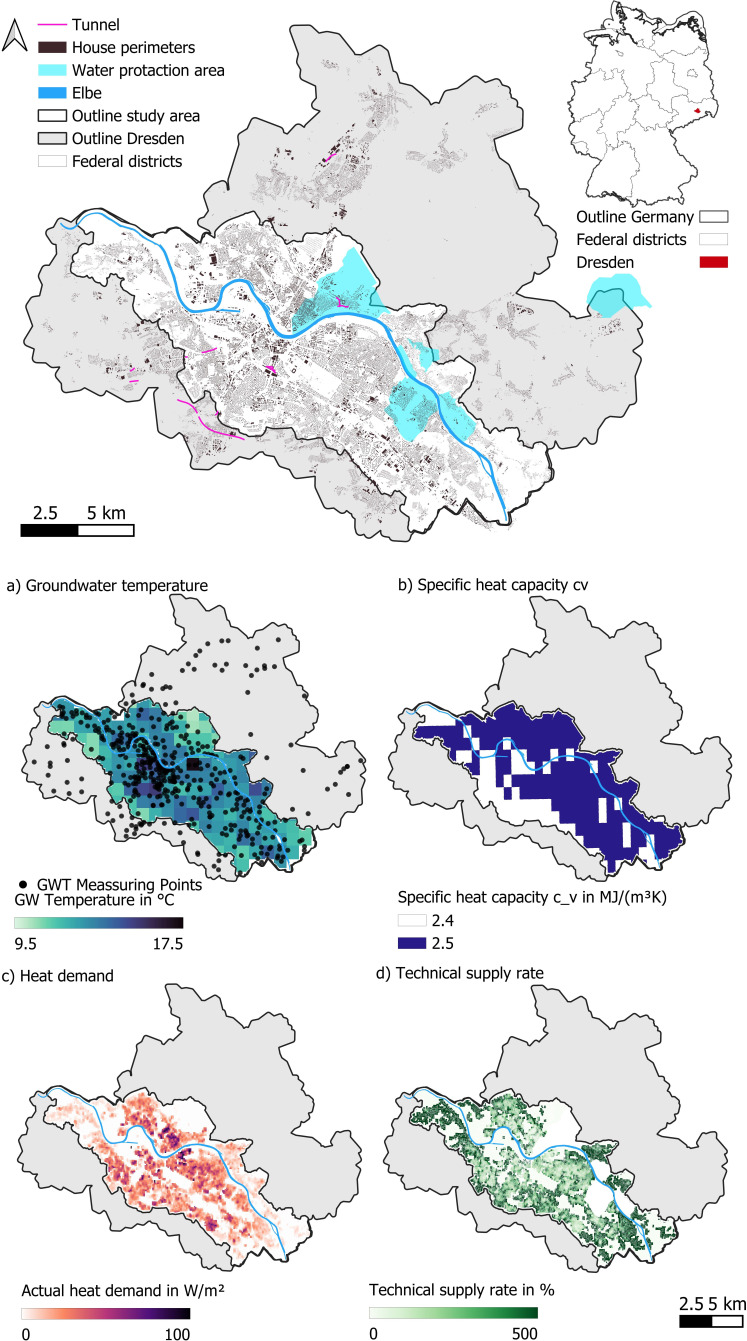
Input data necessary for the evaluation of accumulated heat in our study area in Dresden. (a) GWT in summer 2023 and measuring points, (b) specific heat capacity, (c) residential space HD and (d) technical supply rate.

The analysis and methodology introduced is showcased at the example of Dresden, the state capital of Saxony, Germany. Dresden covers an area of 328.28 km² and is divided into two halves by the river Elbe [34] (for location, area and territory see figure 2). In 2022, nearly 570 000 people lived in Dresden [34]. The mean air temperature in 2024 was 11.7°C. Dresden is rich in groundwater resources. There are essentially two aquifers, which are hydraulically separated from each other. The lower water storage layer consists of Cretaceous and Rotliegend rocks. As the groundwater table is deeper than 100 m below the surface in most parts of Dresden, it can be neglected in the analysis for this study. The upper aquifer is mainly located in the Elbe valley and consists of sands and gravel deposits that are referred to as the Pleistocene main aquifer [35] (water). Groundwater flow is directed towards the river, and the mean upper aquifer depth is 15 m [36]. Owing to a lack of water measurements in the outskirts of the city, the study area is reduced from 328 km${,}^{2}$ to 138 km${,}^{2}$, concentrating on densely built-up areas around the city centre (for more information on Dresden's hydrology, see [37]).

Where possible, analysis is conducted in *Google Earth Engine* [38] to ensure scalability and transferability. For increased readability, all datasets and assumptions used in our calculations are listed in the supplements (electronic supplementary material, tables S1–S3).

### (b) Status quo scenario

#### (i) Accumulated heat supply

The accumulated heat *$Q_{accumulated}$* in MJ per cubic metre in the urban underground is calculated per pixel (10 m × 10 m) for the *status quo* scenario following the methodology introduced in [32]:


(2.1) $$Q_{accumulated}=\Delta T\cdot c_{v},$$

where ΔT is the estimated accumulated heat anomaly and *$c_{v}$* is the mean volumetric heat capacity of the aquifer taken from [32]. We calculate ΔT by subtracting the minimum measured GWT, which is 9.9°C within the study site from local GWT [36]. This value is rather conservative, as the use of a higher initial temperature underestimates the excess heat available for recycling. Still, this GWT value was chosen because, even though we lack rural reference data, a GWT of approximately 10°C can also be observed in the outskirt region of Berlin [29]. Moreover, the aim was to choose a GWT that reflects restored natural habitat conditions, and the choice of a significantly lower starting temperature harbours the risk of creating an urban cold island effect, with potential consequences for the groundwater ecosystem, that is, the opposite to the SubSUHI effect. However, detailed studies are so far missing [39].

GWTs were monitored by the municipality in 473 locations within and outside the Elbe valley. Data were provided as a raster file (figure 2*a*). All measurements were carried out over a period of three weeks between the 12 July and the 4 August 2023, 1 m below the water table [36]. As the depth to the water table varies between less than 2 m and 64 m within Dresden, we must expect depth-dependent seasonal effects in the observed temperature values.

To obtain the *theoretical sustainable* heat supply period, i.e. the number of years that heat can be provided in a sustainable manner, the accumulated heat per m² is divided by the annual residential space HD. Hence, $Q_{acc}$ is multiplied with the mean aquifer depths of 15 m [36], to get heat per m².

HD (figure 2c) is determined from data provided in a 100 m grid by the population and housing census 2022 of Germany [40]:


(2.2) $$HD=hd_{LS}\cdot A,\;$$

where *$hd_{LS}$* is the HD in kWh m^–2^ living space and year, and *A* is the living space in m^2^ living space per m^2^ ground space.

#### (ii) Technical exploitation

To quantify the *technical exploitation* supply rate *S* (figure 2*d*), i.e. the ratio of technically extractable geothermal heating energy and residential HD, we follow the methodology by Tissen *et al.* [21]:


(2.3) $$S_{BHE}=\frac{q_{BHE}\cdot l\cdot n\cdot SPF}{(SPF-1)\cdot HD},$$

where *$q_{BHE}$* is the technically possible extraction rate by BHEs installed at a given depth in the *technical exploitation* case (for values and data see electronic supplementary material, table S1). The mean *technical exploitation* rate of BHE $q_{BHE}$ in our study area is 48 W m^−1^. This depth *l* is limited to 100 m following common standards in Germany [17]. This length has the advantage that responsibility lies solely with the *Lower Water Authority*; *n* is the total number of installable GSHPs. Generally, a minimum distance of 10 m [20] between two geothermal systems is required. Thus, a grid is created where each pixel represents one GSHP installation area of 10 m × 10 m. Excluded for GSHP installation are streets, buildings and water protection areas and minimum distances to infrastructure, such as buildings and streets (2 m) and for tunnels (5.5 m) as shown in figure 2.

Instead of the COP as used by Tissen *et al.* [21], an SPF of 4 [16] is used.

#### (iii) Economic consideration

To better understand the economic feasibility of shallow geothermal heat use, particularly with regard to ${\text{CO}}_{2}$ pricing, we differentiate between two sub-scenarios:

(i) In a *stable* scenario, we assume no changes in prices for gas (0.12 € kWh^−1^ in 2024) and electricity (0.41 € kWh^−1^) [41]. The scenario also assumes constant ${\text{CO}}_{2}$ emission in the electricity mix, i.e. no advances in technology. Following current German laws, the ${\text{CO}}_{2}$ tax of 45 € ton^−1^ ${\text{CO}}_{2}$ (in 2024) is modelled to increase by 10 € ton^−1^ ${\text{CO}}_{2}$ each year until it reaches 65 € ton^−1^ ${\text{CO}}_{2}$ in 2027 [26]. Once this price is reached, we assume no more changes.
(ii) In a *trend* scenario, we assume that gas and electricity prices increase every year by 10.5 [42] and 4.2% [43], respectively. These were the mean linear increases over the last 5 years (2019–2024). We also assume a reduction in ${\text{CO}}_{2}$ emission in the electricity mix of Germany by 0.02 kg kWh^−1^ each year, allowing Germany to meet its goal of ${\text{CO}}_{2}$ neutrality by the year 2045 [24].

In this economic consideration, the number of BHEs required to fully cover the HD of the study area is used. The BHEs are designed according to the *technical exploitation* rate, as this is the current standard design. First, we want to find that point in time at which both systems (geothermal, marked with the subscript geo and traditional gas heating marked with subscript gas) are equally expensive considering investment cost I and operational costs O:


(2.4) $$\begin{array}{r} I_{\text{geo}}+O_{\text{geo}}=I_{\text{gas}}+O_{\text{gas}} \end{array}.$$

Hence, we determine the cost of both systems for increasing values of the running time t, until costs are equal or until the geothermal system is cheaper. If the payback time is less than the usual lifetime of a GSHP of around 25 years, then the system is considered economically advantageous


(2.5) $$\begin{array}{rl} I_{\text{geo}}+\sum_{t=1}^{20}( & \frac{\text{HD}}{\text{COP}}\cdot P_{\text{elec}}\cdot f_{\text{elec}}(t)+E_{{\text{CO}}_{2},elec}\cdot f_{{\text{CO}}_{2},elec}\cdot P_{{\text{CO}}_{2}}(t)\cdot f_{{\text{CO}}_{2}}(t))\\ = & I_{gas}+\sum_{t=1}^{20}(\text{HD}\cdot P_{\text{gas}}\cdot f_{\text{gas}}(t)+E_{{\text{CO}}_{2},gas}\cdot P_{{\text{CO}}_{2}}(t)\cdot f_{{\text{CO}}_{2}}(t)). \end{array}$$

The overall investment costs for the geothermal option are set to 50 000 € per GSHP (including drilling without any subsidy) for a total of 230 000 GSHP. This number is based on the number of BHEs necessary to cover 100% of the HD in our study area using the mean *technical exploitation* potential of Dresden’s underground (48 W m^−1^) (for values and data see electronic supplementary material, table S2) and one BHE per GSHP. This is a very conservative estimate, as it is usually possible to have multiple BHEs supporting one GSHP. For gas heater systems, we calculate 10 000 € per system [44] and the same number of gas heaters as GSHPs. In both scenarios, we assume that the heat demand is fully covered by gas heater systems. In the *trend* case, we assume that 25% of the already installed gas heaters need to be refurbished within the next years as they are older than 30 years and need to be replaced anyway [25].

The operation costs are calculated for all of Dresden as a whole, i.e. HD is summed up over all grid cells in our study area. Operating costs of gas heaters include the gas price (*P*) and the price for the resulting ${\text{CO}}_{2}$ emission ($P_{{\text{CO}}_{2}}$). Operating costs for geothermal systems include electricity costs ($P_{\text{elec}}$) and price for ${\text{CO}}_{2}$ emission based on the electricity mix in Germany. Because gas and electricity prices, as well as the ${\text{CO}}_{2}$ emission (*E*) of Germany’s power mix (for values and data, see electronic supplementary material, table S2) will not stay constant over the next years, we have included a time-dependent factor (*f(t*)) for each of these parameters, to show how increasing prices and reducing ${\text{CO}}_{2}$ emission have an effect on the payback rate. As maintenance costs for both systems are approximately the same, they are not explicitly considered.

We also determine how high a ${\text{CO}}_{2}$ tax would have to be, to reach a payback time of 25 years, i.e. the tax at which price an old gas heater system and a new geothermal system accumulated the same cost after a mean operating time of 25 years. Thus, we change the formula as follows:


(2.6) $$\begin{array}{rl} P_{{\text{CO}}_{2}}(t) & =\frac{I_{\text{geo}}-I_{\text{gas}}+\sum_{t=1}^{20}(\frac{\text{HD}}{\text{COP}}\cdot P_{\text{elec}}\cdot f_{\text{elec}}(t)-\text{HD}\cdot P_{\text{gas}}\cdot f_{\text{gas}}(t))}{\sum_{t=1}^{20}(\text{HD}\cdot E_{{\text{CO}}_{2},gas}-\frac{\text{HD}}{\text{COP}}\cdot E_{{\text{CO}}_{2},elec}\cdot f_{{\text{CO}}_{2},elec}(t))}. \end{array}$$

### (c) Recycled scenario

#### (i) Technical sustainable supply rate

**Figure 3.**
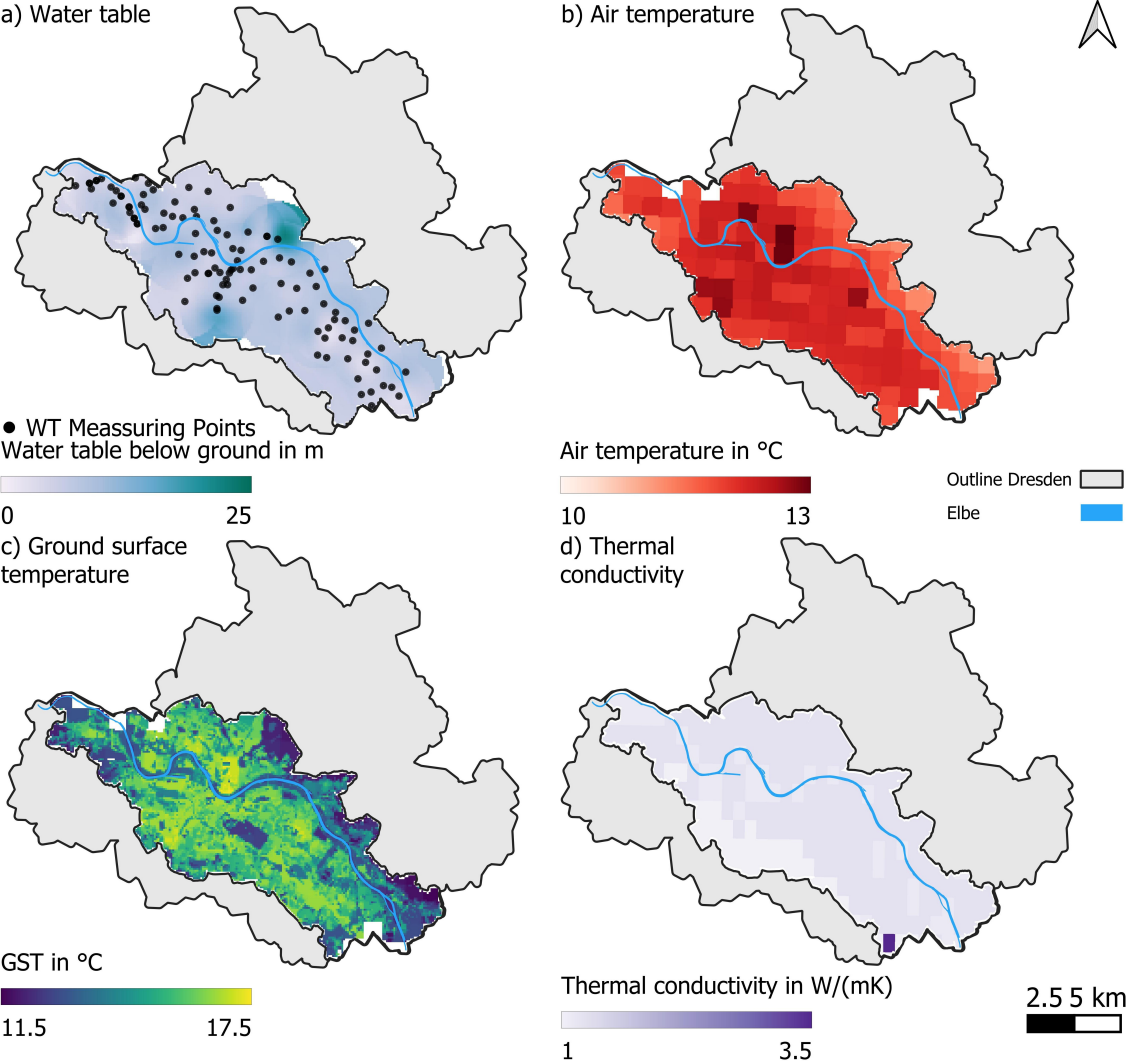
Input data necessary for the evaluation of sustainable supply rate in our study area in Dresden. (a) water table in summer 2021 and measuring points, (b) air temperature, (c) GST and (d) thermal conductivity.

To quantify the annual heat input into the aquifer at its natural state, we focus on heat fluxes caused by buildings, tunnels and the district heating network as well as natural heat fluxes coming from the surface owing to solar radiation and land cover and coming from the earth’s core [21]. All used inputs are listed in electronic supplementary material, table S3. Conductive heat flux *$q_{surf}$* from the surface into the underground is quantified for all areas without buildings and can be quantified as [32]


(2.7) $$q_{surf}=\lambda \frac{T_{surf}-T_{GW}}{D_{GW}},$$

with the thermal conductivity *λ*, ground surface temperature (GST) *$T_{surf}$*, the depth to the water table *$D_{GW}$*, and GWT *$T_{GW}$*. That is, groundwater is at its natural, undisturbed temperature, i.e. the accumulated heat has been fully recycled. In Dresden, this corresponds to the minimum observed value of 9.9°C in the study area [36]. The depth to the water table was observed at more than 100 measuring points within the Elbe valley over a period of three weeks in May and June 2021. We interpolated these values using Gaussian interpolation (figure 3a). GSTs are derived from 10-year mean (2014–2023) air temperatures (figure 3b) available through the *german weather service* as hourly, 1 km resolution raster data [45]. To get GSTs, we add a land use-dependent offset [46] to the air temperatures following the examples of [31] and [21] (figure 3c). Land use information is available from the *European Environment Agency* in their Urban Atlas 2018 [47].

Similar to fluxes from the surface, conductive heat flux *$q_{built}$* from buildings into the underground is quantified for all areas with a building footprint as


(2.8) $$q_{built}=\lambda \frac{T_{built}-T_{GW}}{D_{GW}-D_{built}},$$

with a basement temperature $T_{built}$ and a basement depth $D_{built}$. For values and sources, see electronic supplementary material, table S3.

Fluxes from tunnels *$q_{tunnel}$* are also conductive. Temperatures within them are set to be equal to local air temperatures above ground ($T_{air}$) following the methodology of [21], given that tunnels are generally well ventilated, and any potential underestimation of temperature would lead to conservative (i.e. lower) heat flux estimates. We assume that all tunnels are located below the water table. Hence, tunnel and groundwater are only separated by 0.8 m of wall, and fluxes can be quantified following equations (2.7) and (2.8) with wall thickness as the denominator. For all input values, see electronic supplementary material, table S3.

The district heating network DH is assumed to have constant heat loss *$q_{DH}$* over the whole area of Dresden [31]:


(2.9) $$q_{DH}=\frac{P_{DH}\cdot r_{DH}}{A},$$

with *$P_{DH}$* being the heat loss of the system, *$r_{DH}$* the heat loss directed downwards and *A* the area of city. Again, all values can be found in electronic supplementary material, table S3.

To quantify the total annual anthropogenic heat flux into the urban underground *$q_{tot}$,* the four individual heat fluxes and the upwards directed natural heat flux *$q_{uw}$* are summed to get the total heat flux *$q_{tot}$*. The total annual heat flow *$Q_{annual}$* in Wh per year of the entire study area is then calculated by summing up all pixel cell values.

Similar to the *technical exploitation* supply rate in the *status quo* scenario, we now define the *technical sustainable* supply rate *SusS* as the ratio of *$Q_{annual}$* that can be extracted in a sustainable manner and the annual HD


(2.10) $$SusS_{BHE}=\frac{Q_{annual}\cdot SPF}{(SPF-1)\cdot HD}.$$

The result represents the *technical sustainable* supply rate *SusS* of Dresden’s shallow subsurface, taking into account the electricity that is needed to harness the sustainable geothermal potential. Using the *SusS* for HD coverage means installing more GSHPs than considered in §2b(iii), as the technical exploitation rate is not fully used.

We look at the *technical sustainable* potential for the entire city under three different cases:

(i) The *technical sustainable* case: Here, we calculate the city-wide technical sustainable supply rate if we used all possible installation areas (cf. figure 2) for BHEs and only extracted the annual sustainable heat input. That is, the average *SusS* for all 10 m × 10 m grid cells is calculated.
(ii) The 100% coverage case: Here, we calculate the maximal HD coverage if we only installed BHEs in areas where the *technical sustainable* supply rate is at least 100%, because these systems make the most economic sense. That is, *SusS* for all grid cells where it is smaller than one is set to zero before the citywide average is calculated.
(iii) The 65% coverage case: In this case, we calculate the maximal HD coverage if we installed BHEs in areas where the *technical sustainable* supply rate is at least 65% as this is what the *German Buildings Energy Act* [25] states: every new installed heating system needs to run with at least 65% renewable energies. That is, SusS for all grid cells where it is smaller than 0.65 is set to zero.

For the second and third cases, we also calculate how many people would benefit from BHE installations.

#### (ii) Social consideration

We aim to determine whether there are significant disparities in access to sustainable heat supply through shallow geothermal heat recycling. Particularly, whether vulnerable populations—such as the elderly, children or economically disadvantaged individuals—have equal access to sustainable heat than their advantaged neighbours.

To analyse this, we link our results to socio-economic populations mapped by the 2022 population and housing census [40] in a 100 m grid (for more information, interactive maps can be found in [48]) and assess the distribution of sustainable heat supply (*SusS*) across Dresden’s overall population and by districts individually. We examine the following socio-economic data:

(i) Net rent in € per m²
(ii) Share of property owners in per cent
(iii) Share of foreigners in per cent
(iv) Living space in m² per person
(v) Share of individuals under 18-years-old in per cent
(vi) Share of individuals over 65-years-old in percent

To understand which socio-economic groups may benefit from heat recycling, we first test for trends between census data and *SusS* for all districts in our study area (electronic supplementary material, fig. S1). We only include districts that are covered fully by our study area (i.e. districts one to eight). We determine a linear trend over all districts combined and for each district individually.

### (d) Future scenario

#### (i) Sustainable supply rate with climate change

In our *future* scenarios, we repeat what was done for the *recycled* scenario in §2c, substituting air temperature and thus GST with projections for 2100. In addition, heat input from the district heating network is eliminated as we assume it will be discontinued once the heat-providing industry turns carbon neutral. For the projections, we follow a medium emissions scenario (Shared Socio-economic Pathway SSP 245) and a worst-case scenario (SSP 585) of the *CMIP6* (*Coupled Model Intercomparison Project*) [49] as suggested by the IPCC [50]. Owing to a lack of data, we assume no changes in HDs and land cover.

## 3. Results and discussion

### (a) Status quo scenario

#### (i) Accumulated heat supply

**Figure 4.**
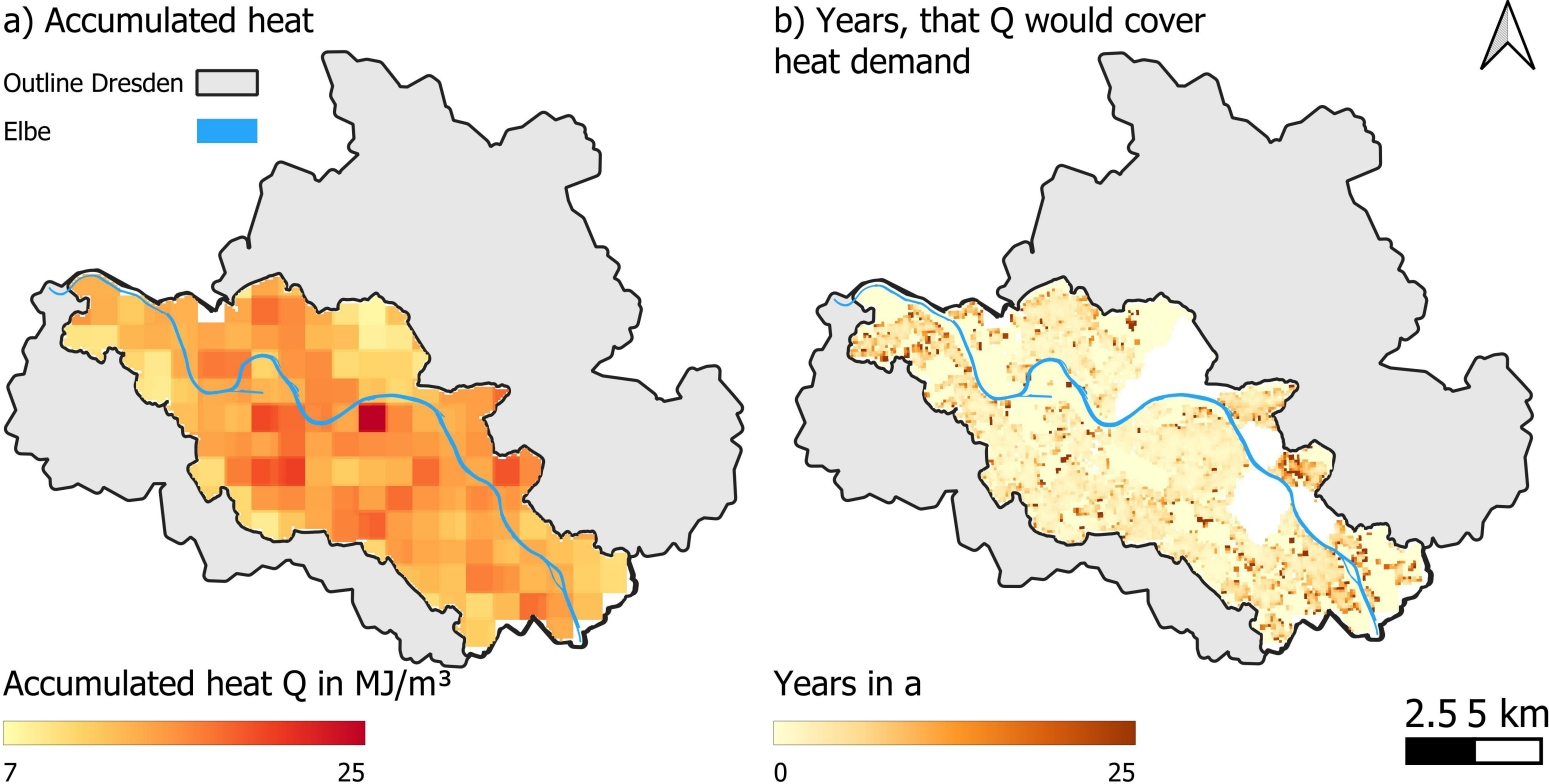
Results of the evaluation of heat fluxes in our study area in Dresden. (a) accumulated heat and (b) how many years the accumulated heat could cover the residential HD considering the technical extraction rate.

The mean accumulated heat (*$Q_{accumulated}$*) in the urban aquifer is 13.9 MJ m^−3^ (figure 4a). On average, if we do not consider any limitations on heat extraction and transportation nor political, financial or social limitations, the accumulated heat could cover residential HD for approximately 3 years. However, spatial variations exist (figure 4b). Most of the heat has accumulated in the city centre, as this is the oldest part of the city and human activity and the resulting heat fluxes have had more time to accumulate underground. However, owing to the high population densities in the city centre, residential HD is also highest. Accordingly, the accumulated heat would only last approximately 3 years. In the outer areas of Dresden, the accumulated heat could cover the HD for 10 to over 25 years, which meets the lifetime of a GSHP. Lower residential HD and more available space for the extraction of geothermal heat in the outer districts ‘compensate’ for less accumulated heat in the underground. After these times, groundwater would have cooled down until reaching a stable temperature, and the SubSUHI would be gone. At this point, we enter the *recycled* scenario presented in §2c.

#### (ii) Technical exploitation

If the *technical exploitation* rate is considered, around 230 000 BHE would currently be required for the study area to cover residential HD. As the installation of one GSHP with two BHEs takes about two weeks and as the German energy transformation is scheduled to be done in 20 years (by 2045), 110 heat pumps would have to be installed per week. In particular, considering the current shortage of skilled workers and bottlenecks in delivery, this seems utopic. In reality, however, the conversion of the heating market to 100% heat coverage has to be achieved through various methods of renewable energy generation, as the pure use of geothermal systems would exploit the ground and would lead to undercooling and even freezing [17] and reverse the effect of today’s overuse, which heats up the ground.

#### (iii) Economic consideration

For the economic feasibility of shallow geothermal heat recycling, we consider the two cases described in §2biii.

**Figure 5.**
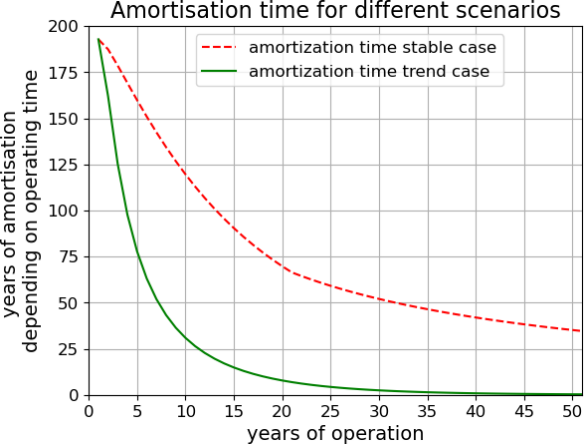
Payback time for different scenarios.

In the *stable* case (i.e. no changes in tax, gas price and electricity mix), the payback time for heating all of residential Dresden using geothermal systems is more than 41 years (figure 5, dashed line). To achieve a fair balance within a 25-year system lifetime, the ${\text{CO}}_{2}$ tax would have to be set at 790 € ton^−1^ of ${\text{CO}}_{2}$, which is more than 12 times higher than the regulated maximal ${\text{CO}}_{2}$ pricing of 65 € ton^−1^ of ${\text{CO}}_{2}$. Accordingly, in a *stable* case, the current ${\text{CO}}_{2}$ pricing is too low to make a citywide GSHP system economically advantageous to pre-installed heating systems. However, current ${\text{CO}}_{2}$ pricing is not a true reflection of the actual cost of carbon [51,52]. A fair price, which accounts for the damages caused by ${\text{CO}}_{2}$ emissions, would range between 300 and 880 € ton^−1^ of ${\text{CO}}_{2}$ as calculated by the *Federal Environment Agency* [51]. This would make citywide GSHPs economically feasible (if not practically as described above). Even more so if we consider the *IPCC 5* estimates aiming at a below 1.5°C pathway [52] as internationally agreed on in the Paris Agreement [27]. They suggest 128–5770 € ton^−1^ on ${\text{CO}}_{2}$ in 2030, increasing in the future.

This shows that if we priced all environmental damages to the ${\text{CO}}_{2}$ price, even in the *stable* case, the savings in operation costs would mask the higher investment costs of GSHP during a system's lifetime.

We also include a *trend* case which assumes favourable but still realistic changes in gas and electricity pricing, as well as investment costs for refurbishment of gas heaters. In this case, the payback time for the system is approximately 15 years (figure 5, continuous line), even if the ${\text{CO}}_{2}$ price does not exceed 65 € ton^−1^. This means that the average price increase over the last 5 years of ${\text{CO}}_{2}$ price (and other taxes) for electricity and gas would be sufficient if it were to develop in the same way in the future and if Germany reaches its climate-neutrality goal in the electricity market by 2045.

### (b) Recycled scenario

#### (i) Sustainable supply rate

**Figure 6.**
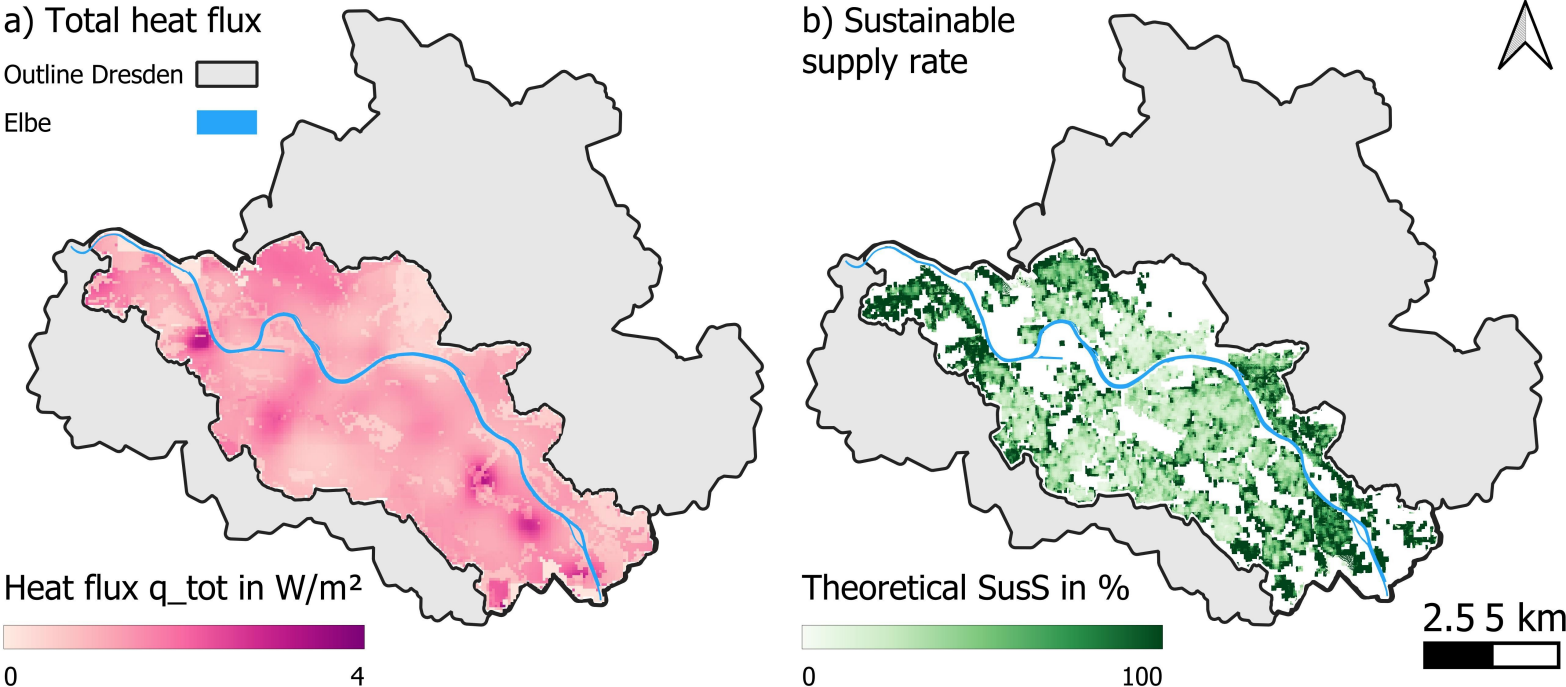
Results of the evaluation of heat fluxes and *SusS* for the recycled scenario in our study area in Dresden. (a) Total heat flux and (b) *theoretical sustainable* supply rate.

In the *recycled* scenario, the accumulated heat has been recycled and GWTs are at a stable level of 9.9°C. A map of the total heat input to the urban underground can be found in figure 6a, and is on average 1 W m^−2^. The heat input from the surface owing to elevated GST is the highest (average value is 0.62 W m^−2^), followed by the heat input from buildings (average value is 0.16 W m^−2^). As shown in previous studies, the highest heat fluxes are found in locations with a very shallow water table, i.e. less than 3 m below the surface [31]. As our study looks at the *recycled* scenario, we cannot compare our results to other studies that calculated the heat fluxes for the *status quo* only.

Figure 6b shows a map of the *SusS* per 100 m grid cell. We find that the residential HD could only be covered in a sustainable manner in the outer areas of Dresden (i.e. a *SusS* of greater than 1). This is due to the low HD and low population density combined with sufficient space for installing BHEs. This fits the results for Vienna [21].

**Figure 7.**
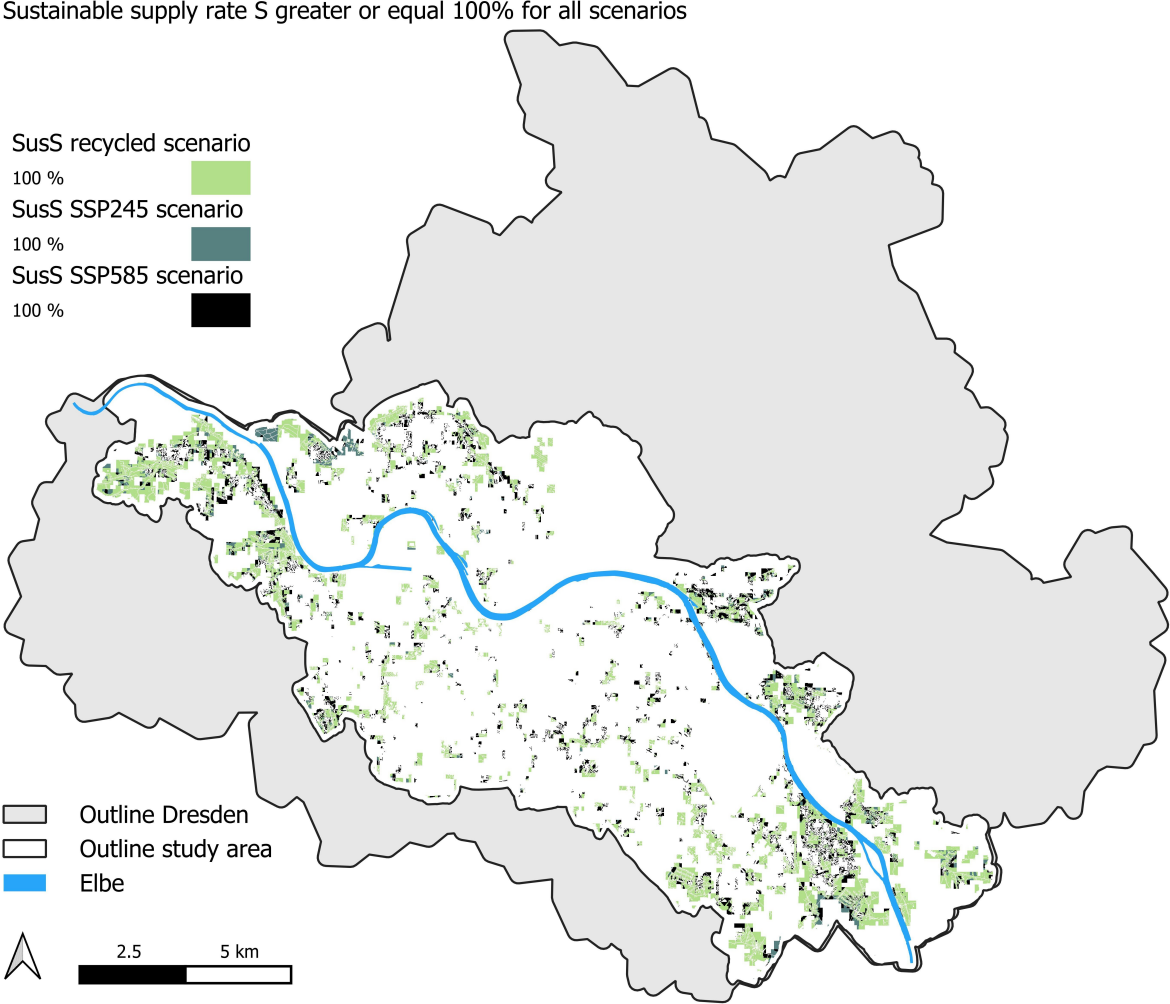
Sustainable supply rate (*SusS*) for recycled and future scenarios.

(i) In the *theoretical sustainable* case, all heat would be extracted in a sustainable manner (e.g. using the annual heat input *q-tot* as extraction rate) and without taking into account limitations on heat transportation within a pixel, we could cover approximately 47% of Dresden’s HD (figure 6b), but we would need more than 850 000 BHEs, which is not economical.
(ii) In the *technical sustainable* case, we would install GSHP at places where the *SusS* would cover at least 100% of the local residential HD. Approximately 2.3% of the total residential HD in our study area could be supplied in a sustainable manner (figure 7, *recycled scenario*). Approximately 21 300 people would benefit from this.
(iii) If we take into account the *German Buildings Energy Act* [25], which states that every newly installed heating system needs to run with at least 65% renewable energies, this would increase the possible coverage of HD in Dresden to approximately 4.5% in the recycled scenario, which would benefit 45 000 people for the *technical sustainable* case.

The sustainable extraction rates could significantly increase in all cases if the potential for geothermal cooling were considered. Using GSHP for both heating and cooling not only improves system efficiency but also helps balance subsurface energy use by storing heat in summer for use in winter. This can broaden the economic and ecological benefits, e.g. reduced ${\text{CO}}_{2}$ emissions from conventional cooling.

#### (ii) Social consideration

Electronic supplementary material, fig. S2 shows a comparison between net rent per person and the sustainable supply rate in each 100 m grid cell. When looking at all districts at once, no trend in either direction is visible. A sustainable supply rate approximately 2.5% is given over all net rent prices that range from 5 € to 9 € m^−2^. The individual analysis per district (electronic supplementary material, fig. S3) shows that the direction of the trend is inhomogeneous (positive relation in districts Altstadt, Blasewitz, Leuben, Prohlis, Plauen; negative relation for Neustadt, Pieschen and Cotta) and none is significant. The negative trends are all in rather rural districts, with the highest *SusS* particularly at their outskirts. Here, rents are low owing to the comparatively bad connection to town.

Electronic supplementary material, fig. S2 shows a comparison between property owner rate and the sustainable supply rate. A positive relation is visible but not significant with a coefficient of determination *R*² of 0.3. In the individual analysis (electronic supplementary material, fig. S4), all districts show a positive relation with *R*² from 0.1 up to approximately 0.7. In particular, the districts of Altstadt, Neustadt and Prohlis show significant trends (*R*² > 0.6). That is, there, heat recycling is particularly feasible in areas where the inhabitants own the ground they are living on and thus have the rights to apply for the installation of GSHPs if they choose to do so.

Electronic supplementary material, fig. S2 shows a comparison between the share of foreigners and the sustainable supply rate. In the overall perspective and in most districts (electronic supplementary material, fig. S4), there is a slight negative trend (i.e. increase in foreign inhabitants with decreasing *SusS*); however, none are particularly significant or even notable. Electronic supplementary material, fig. S2 shows a comparison between living space per person and the sustainable supply rate, which is neither significant in the overall nor in the individual analysis (electronic supplementary material, fig. S3). We also compared the share of over 65-year-olds and the sustainable supply rate but could not find any relevant conclusion. These data did not show any significant trend for either the overall (electronic supplementary material, fig. S2) or individual analyses (electronic supplementary material, fig. S3). The same applies to the share of under 18-year-olds (electronic supplementary material, figs. S2 and S4).

### (c) Future scenario

#### (i) Sustainable supply rate with climate change

In this section, we discuss the sustainable supply rate *SusS* in the *future* scenario.

(i) In the *theoretical sustainable* case, all sustainable heat will be used without limitations of heat extraction rates (economic costs) and heat transfer from site to building. We could achieve a coverage of 51.8% of the HD in *SSP245*, and up to 81.7% HD coverage in the *SSP585* scenario. As residential HD is projected to reduce in the future (owing to warming temperatures and better insulation), our results represent a conservative estimate. This underlines that the potential for geothermal energy increases as a result of climate change, as was predicted in [32]. So, climate change clearly has an effect on sustainable geothermal energy, and the higher the air temperature, the higher the heat fluxes and hence higher sustainable supply rates.
(ii) In the *technical sustainable* case, BHEs are installed only in locations where the annual heat flux can fully cover the HD. The *technical sustainable* potential would be sufficient to cover 2.7% of the HD in the *SSP245* and 4.5% in the *SSP585* scenarios. Around 24 000 people would benefit from this in the *SSP245* scenario, and around 400 000 people in the *SSP585* scenario, which is around 10% of the study area population. The areas that cover 100% of the required HD in that area are shown in figure 7 for all scenarios.
(iii) If HPs are installed in locations where the annual heat flux can cover the HD of at least 65%, the *technical sustainable* potential would be sufficient to cover 5.0% (nearly 50 000 people) of the HD in the *SSP245* and 7.6% (close to 75 000 people) in the *SSP585* scenarios, respectively.

However, if shallow geothermal systems are widely adopted, they may contribute to a reduction in ${\text{CO}}_{2}$ emissions and thus potentially mitigate climate change. In turn, this could lead to a decrease in heat fluxes and consequently reduce the amount of excess heat available for recycling.

## 4. Conclusion

In this study, we described a timeline for shallow geothermal heat recycling at the city scale at the example of Dresden, Germany. Starting in a *status quo* scenario, we quantified the accumulated waste heat in the urban underground. If the accumulated heat is extracted, it would cover the cities' residential space HDs for more than 3.5 years, after which the GWT would return to a baseline temperature consistent with the surrounding area. However, this heat extraction rate is not distributed equally, and infrastructure to transport it within the city limits would be necessary. Furthermore, even in the *technical exploitation* scenario, 230 000 numbers of BHE would have to be installed to meet the HD, which is not economically feasible. Still, this analysis reveals that subsurface waste heat accumulation in Dresden should be considered a resource and taken into consideration for future construction.

We also evaluated the economic potential of heat pumps outside their sustainability. We find that a transformation of the heating market from gas heaters to geothermal systems would not pay off with current ${\text{CO}}_{2}$ tax and *stable* price conditions. Under *trend* conditions, however, the high investment costs of GSHP systems would compensate for the high operation cost of gas heaters long before the end of system lifetime of 25 years. From an ecological–economical perspective, environmental damages should always be considered when comparing heating systems and could reduce the payback time to less than 25 years even under *stable* conditions. This means that by implementing ${\text{CO}}_{2}$ pricing through the *German Fuel Emissions Trading Act* [26], which eliminates fossil fuel subsidies by accounting for ${\text{CO}}_{2}$-related damages, and by enhancing the share of renewable energy in the electricity mix as outlined in the *Federal Climate Action Act* [24], the legal framework for a fair and sustainable transformation of the heating market is firmly established and on the right track.

Once the accumulated heat has been recycled, we enter a scenario where sustainable shallow geothermal usage has to be fueled through annual heat input. Natural and anthropogenic heat fluxes in the urban area are visualized for the *recycled* scenario and indicate that outer areas of the city are particularly well suited for sustainable shallow geothermal heat recycling. Owing to low HDs, both accumulated heat in the *status quo* scenario and the annual incoming heat flow in the *recycled* scenario can cover residential needs. This potential would further increase in the *future* scenarios, where the heat input from the surface into the subsurface is particularly high. However, in these regions, shallow geothermal heating systems do not benefit from the efficiency boost of SubSUHI in the *status quo*.

On the other hand, people living closer to or in the city centre have less benefit from sustainable energy per person. While there is more accumulated heat and more heat input in total, it does not meet the need of all people. To supply the demand of all people, the *technical exploitation* potential needs to be extracted. This is not necessarily sustainable and would cool down GWT even further than the pre-industrial level. Else, it has to be decided who gets to benefit from the extracted heat.

By comparing the sustainable supply rate to socio-economic data, we tested for occurrences of energy (in)justice. However, in part owing to the small study site, no decisive conclusions could be made. As mentioned above, populations living in the city centre are at a disadvantage compared to populations at the outskirts of town.

However, this study does not include the potential for geothermal cooling in Dresden, which could balance subsurface energy use, enhancing sustainability by minimizing the risk of overexploiting (freezing) the ground. Shallow geothermal heat pump systems become more efficient when used for both heating and cooling, while only requiring a single system to manage both processes, simplifying infrastructure and reducing costs. Cooling with geothermal systems in summer insert heat into the ground, where it is stored. This stored heat can then be extracted in winter to provide heating. This extra anthropogenic heat flux can help to increase the sustainable supply rate and therefore help maximize the societal benefit. Moreover, the implementation of geothermal cooling could amplify ecological benefits by reducing ${\text{CO}}_{2}$ emissions compared to conventional air conditioning systems. Hence, everyone would benefit, not only the users of geothermal energy.

## Data Availability

All codes and data sources are available at Zenodo [53]. Supplementary material is available online [54].
